# Functional Spermatogenesis Across Testicular Developmental Stages in Neomale Large Yellow Croaker (*Larimichthys crocea*) Revealed by Histology and Gonadal Specific Cellular Markers

**DOI:** 10.3390/biology14081054

**Published:** 2025-08-14

**Authors:** Xu Liu, Weihua Hu, Ruiyi Chen, Yang Yang, Sixian Yang, Dongdong Xu

**Affiliations:** 1 Fisheries College, Zhejiang Ocean University, Zhoushan 316022, China; bahkyyn1@163.com (X.L.); 15855771489@163.com (S.Y.); 2 Key Laboratory of Mariculture and Enhancement, Zhejiang Marine Fisheries Research Institute, Zhoushan 316021, China; huweihua@webmail.hzau.edu.cn (W.H.); xx_cry@163.com (R.C.); yangyang19911105@163.com (Y.Y.)

**Keywords:** spermatogenesis, testicular development, immunofluorescent, neo-males, *Larimichthys crocea*

## Abstract

Gonadal development and spermatogenesis critically influence fish reproductive performance. Neomales (genetically female but functionally male) are indispensable for generating all-female populations, yet their spermatogenesis remains understudied. In the present study, we systematically investigated gonadal maturation in neomales of the large yellow croaker (*Larimichthys crocea*), an economically important marine species exhibiting sexually dimorphic growth. We examined the growth performance and gonadal development throughout the maturation process in neomales and control males. Results showed comparable growth performance but a temporal divergence in gonadal development: the gonadosomatic index (GSI) of neomales was significantly higher than control males at 400 and 430 days post-hatching (dph), but not at 460 dph during the reproductive period. Histological, ultrastructural (TEM), and immunofluorescence analyses collectively demonstrated that neomale testes contained all major spermatogenic cell types. Their morphological characteristics and expression patterns of key markers—germ cells (*vasa*), Sertoli cells (*sox9a*), and meiotic recombination (*dmc1*)—were similar to control males. These findings enhance understanding of gonadal development and spermatogenesis in neomales, providing a theoretical and technical foundation for large-scale production of all-female large yellow croaker.

## Simple Summary

The large yellow croaker (*Larimichthys crocea*) is an economically important fish species in China, with females growing significantly faster than males. As broodstock for all-female production, spermatogenesis in neomales critically determines the quality and quantity of all-female offspring, thus impacting the stability of large-scale mono-sex production. However, studies on spermatogenesis in neomales remain scarce. Here, we compared neomales with normal males of the same age, analyzing growth performance during gonadal maturation and systematically evaluating spermatogenesis. Results revealed that neomales exhibited comparable growth rates, spermatogenic progression, testicular morphology, and expression profiles of key proteins to those of normal males. This discovery advances understanding of gonadal development and spermatogenesis in neomales, supporting large-scale production of all-female large yellow croaker.

## 1. Introduction

The large yellow croaker (*Larimichthys crocea*) stands as one of China’s most economically significant marine fish species. The aquaculture practices for this species have advanced rapidly in recent years, and play a crucial role in seafood supply [1]. However, the industry faces persistent challenges including slow growth rates, disease susceptibility, and precocious puberty that hinder sustainable development [2]. These issues underscore the urgent need for innovative breeding strategies to ensure industry sustainability. Due to the sexual growth dimorphism (females exhibit a 26% faster growth rate than males by the age of 25 month [3]), research efforts have focused on mono-sex breeding approaches, primarily focusing on early gonadal differentiation [4], gynogenesis [5,6] and development of sex-specific molecular markers [7,8]. Significant progress has been achieved in sex reversal, with successful induction of neomales (sex-reversed females) through letrozole (LTZ) oral administration. This breakthrough has enabled the mass production of all-female populations in large yellow croaker aquaculture [9].

Neomales, as artificially induced sex-reversed individuals, are pivotal for establishing all-female populations, as these populations can be produced on a mass scale by crossing neomales with normal females [9,10,11]. The gonadal development and spermatogenesis in neomales directly influence the reproductive performance and yield of all-female offspring [12]. Spermatogenesis is an essential process in male fish reproduction, involving the proliferation and differentiation of spermatogonial stem cells to form mature sperm, thereby ensuring genetic diversity and reproductive success [12]. Proper spermatogenic progression is essential for generating functionally competent sperm, and disruptions at any stage can impair the formation of mature sperm [13]. Therefore, elucidating the characteristics of gonadal development and maturation in neomales is fundamental for assessing their reproductive potential and ensuring the stable production of all-female populations.

To date, the production of neomales through aromatase inhibitor treatment has been achieved in various teleost species, including yellow catfish (*Pelteobagrus fulvidraco*), hybrid red tilapia (*Oreochromis* spp.), rosy barb (*Pethia conchonius*), and three-spot wrasse (*Halichoeres trimaculatus*) [14,15,16,17]. The effects of aromatase inhibitors on gonadal development demonstrate significant interspecies variations, manifesting as diametrically contradictory outcomes across different fish species. Aromatase inhibitor treatment promotes spermatogenesis in yellow catfish, three-spot wrasse, and common carp (*Cyprinus carpio* L.) [14,17,18]. In contrast, the treatment by aromatase inhibitor demonstrates no observable impact on spermatogenesis in European sea bass (*Dicentrarchus labrax*) or black sea bass (*Centropristis striata*) [19,20]. Notably, divergent effects emerge in rosy barb and hybrid red tilapia, where aromatase inhibitors induce structural disorganization of testicular architecture and morphological anomalies in spermatogenic cells, ultimately impairing normal spermatogenesis [15,16]. These contradictory findings underscore the necessity for systematic investigations into neomale gonadal development in different species.

Despite concentrated research efforts on neomale induction protocols in large yellow croaker, the comprehensive characterization of gonadal development and reproductive maturation dynamics in neomale specimens remains undocumented. The knowledge gap that severely constrains commercial-scale propagation of all-female populations for mono-sex aquaculture systems. In the present study, the developmental trajectories of letrozole-induced neomales in large yellow croaker were investigated through multiple approaches integrating histological observations, transmission electron microscopy (TEM)-based ultrastructural profiling, and immunofluorescence staining of germline biomarkers (Vasa, Sox9a, DMC1). Through comparative analysis of gonadal structure between neomales and control males, this study develops a framework to assess neomale reproductive capacity. These findings provide insights into the gonadal development of the sex reversal fish and delineate operational frameworks for optimizing mono-sex breeding protocols in commercial aquaculture systems.

## 2. Materials and Methods

### 2.1. Ethics

The animal study protocol was approved by the Committee on Ethics of Animal Experiments at Zhejiang Ocean University (protocol code 2024053, approved on 19 March 2024).

### 2.2. Fish

A total of 92 fish used in this study were obtained from the research station of Zhejiang Marine Fisheries Institute (Xixuan Island, Zhoushan, China). Neomales were generated through letrozole (LTZ, AdooQ Bioscience, Nanjing, China, A10523) treatment following the protocol described by Mu et al. (2024) [9]. Fry from a genetically uniform batch were acclimated in fiber-reinforced plastic tanks from 40 to 45 days post-hatching (dph), then fed commercial pellets containing 10 mg/kg LTZ from 45 to 125 dph. Following the 80-day treatment, neomales were dorsally labeled with green fluorescence and cultured together with orange-labeled control males for subsequent experiments. Both groups originated from the same fry batch to ensure genetic uniformity.

Fin clips were collected for DNA identification, and the genotypic sex was determined using a sex-specific molecular marker [7]. The gonads were dissected and fixed in Bouin’s fixative. The phenotypic sex was identified by observing histological sections under a microscope. Individuals exhibiting XX genotype yet developing functional male gonads were designated as neomales.

### 2.3. Sampling

Sampling was conducted at 150, 180, 240, 300, 360, 400, 430, and 460 dph. The fish were anesthetized with MS-222 prior to sampling. At each sampling interval, the body length (BL) and body weight (BW) of every individual were measured and recorded. The gonad of each individual was weighted and GSI (gonad weight/body weight × 100) was calculated at 360, 400, 430, and 460 dph. For each sampling time point, the number of fish, tissues collected, preservation conditions, and experiments conducted are detailed in Supplementary Materials Table S2.

### 2.4. Gonadal Histology

The gonads fixed in Bouin’s solution were then transferred to 70% ethanol, dehydrated through a graded ethanol series, embedded in paraffin, and sectioned into 5 μm-thick slices. The slices were stained with hematoxylin and eosin (H&E) for histological observation. Eight fields of view were observed for each slice of each fish. Based on conventional research practices, the developmental stages of the testis are defined according to the predominant germ cell types present within the testis: Stage II: Predominantly spermatogonia. Stage III: Predominantly spermatocytes. Stage IV: Containing various types of germ cells at different developmental stages, with a small quantity of sperm appearing within the lobular lumen. Stage V: Characterized by a large quantity of sperm within the lobular lumen [21]. The sections were examined and photographed using a light microscope (model Axio Imager A2; Zeiss, Gottingen, Germany).

### 2.5. Transmission Electron Microscopy (TEM) Analysis

The dissected gonads were cut into fragments (<1 mm) and immersed in 2.5% glutaraldehyde Fixative in 1 × PBS (pH 7.4) at 4 °C for 24 h. Then, the gonads were washed with 1 × PBS, fixed with 2% osmium tetroxide (OsO_4_) at 4 °C for 4 h, and dehydrated using a graded series of ethanol (30, 50, 75, 95, 100, and 100%) for 10–20 min each. Subsequently, they were embedded in Epon-Araldite for subsequent ultrathin sectioning. Ultrathin sections (75–80 nm) were cut using the Ultracut E ultramicrotome (Leica Microsystems, Vienna, Austria), stained with UranyLess EM stain, counter-stained with 1% lead citrate, and examined under a JEM-1400Flash transmission electron microscope (JEOL, Akishima, Japan).

### 2.6. Immunofluorescent Analysis

Testicular tissues were fixed in 4% paraformaldehyde (PFA) overnight at 4 °C, dehydrated through a graded ethanol series, embedded in paraffin wax, and sectioned at 5 μm thickness. Following deparaffinization in xylene and rehydration through graded ethanol, antigen retrieval was performed by incubating sections in 1 × Universal Powerful Antigen Retrieval Solution (Biosharp, Hefei, China, BL703A) at 95 °C for 30 min. After cooling to room temperature, sections were blocked with 10% goat serum in PBS for 2.5 h at room temperature. Primary antibodies were applied overnight at 4 °C: rabbit anti-VASA (1:1000; Abcam, Cambridge, UK, ab209710), rabbit anti-Sox9a (1:1000; Abcam, Cambridge, UK, ab209820), and rabbit anti-DMC1 (1:300; ABclonal, Wuhan, China, A4491). Sections were then washed five times in PBS (10 min per wash) and incubated with goat anti-rabbit IgG Alexa Fluor^®^ 488 secondary antibody (1:2000; Abcam, ab150077) for 1 h at 37 °C. After three additional PBS washes (5 min each), nuclei were counterstained with DAPI (10 μg/mL; Biosharp, Hefei, China, BL105A) for 5 min at room temperature. Finally, slides were mounted and imaged using an Axio Imager A2 microscope equipped with an Axiocam 506 camera (Zeiss, Gottingen, Germany).

### 2.7. Statistical Analysis

All the data were presented as means ± standard error of the mean (SEM) using GraphPad Prism 10.0 software. Significant differences in BL and BW were determined using one-way ANOVA followed by the Kruskal–Wallis multiple comparisons test (*p* < 0.05). Significant differences in GSI and qPCR were determined using the Student’s *t*-test (Mann–Whitney test) (*p* < 0.05).

### 2.8. Quantitative Real-Time PCR (qPCR)

Testicular tissues were collected from two groups (neomales and control males) at specified developmental stages: Stage II (360 dph), Stage III (400 dph), Stage IV (430 dph), and Stage V (460 dph). Total RNA was extracted from the collected testicular samples using the RNA extraction kit (Solarbio, Beijing, China). The expression patterns of key spermatogenesis-related genes (*vasa*, *sox9a*, *dmc1*) were analyzed by quantitative real-time PCR (qPCR). The specific primers of the genes were designed using Primer Premier 5.0 and listed in Supplementary Material Table S1. *β-actin* and 18 s were chosen as the reference genes. Total RNA was extracted from ovaries and testes using the RNA extraction kit (Solarbio, China). The first-strand cDNA was synthesized using a Transcript First-strand cDNA Synthesis Kit (TranStart, Beijing, China). The qPCR amplifications were performed using 2 × SYBY Premix Ex TapTM II (Takara, Dalian, China) according to the manufacturer’s instructions. PCR amplification was performed using a reaction volume of 20 μL, with 6.8 μL ddH_2_O, 0.4 μL forward and reverse primers (10 μmol/L), 2 μL cDNA, 0.4 μL ROX Reference Dye I and 10 μL 2 × SYBY Premix Ex TapTM II. The PCR reaction was performed under the following conditions: 30 s at 95 °C; 5 s at 95 °C and 30 s at 60 °C for 33 cycles; finally, 15 s at 95 °C, 30 s at 60 °C, and 15 s at 95 °C was used for the dissociation stage. Each assay included a no-reverse transcriptase and a no-template control. The relative gene expression levels were analyzed using the $2^{-\Delta \Delta CT}$ method.

## 3. Results

### 3.1. Growth and GSI

**Figure 1:**
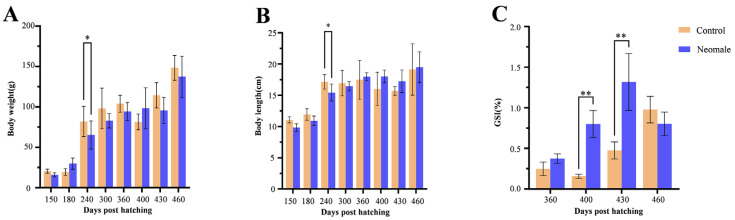
Growth and gonadosomatic index (GSI) of neomale and control male large yellow croaker (*Larimichthys crocea*) from 150 to 460 days post-hatching (dph). (**A**) Body length; (**B**) body weight; (**C**) gonadosomatic index (GSI). Values are considered to be significantly different among groups at *p* < 0.05 (*) and *p* < 0.01 (**). Error bars represent standard deviation (SD).

**Figure 2:**
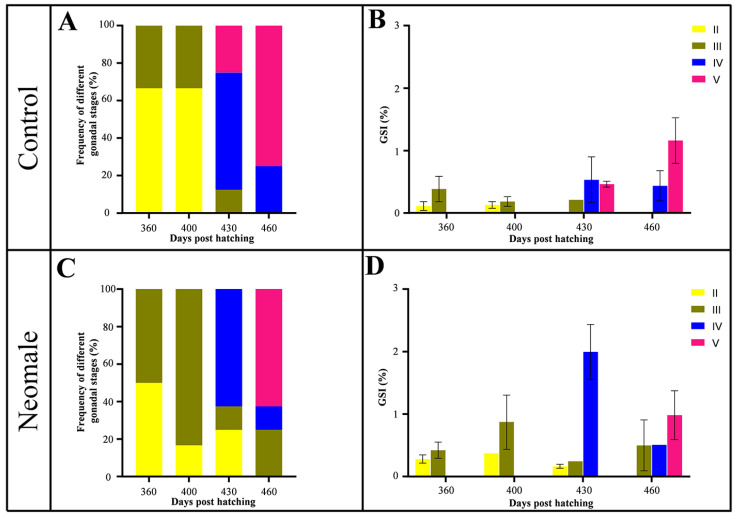
Gonadal stage frequency and GSI in large yellow croaker (360–460 dph). Control males: (**A**) stage frequency distribution; (**B**) GSI across stages. Neomales: (**C**) stage frequency distribution; (**D**) GSI across stages. Error bars represent standard deviation (SD).

The growth data of neomales and control males were compared from 150 to 460 dph. No significant differences in body weight (BW) and body length (BL) were observed at most sampling time points, except at 240 dph, where statistically significant differences were detected. The gonadosomatic index (GSI) was measured in both neomales and control males at 360, 400, 430, and 460 dph (Figure 1C). To investigate the dynamics of GSI during gonadal maturation, we quantified GSI values at each sampling time point (Figure 2B,D). In neomales, the average GSI exhibited an initial increase followed by a decline, peaking at stage IV testis at 430 dph (Figure 1C and Figure 2D). In contrast, control males displayed a consistent upward trend in GSI, reaching their maximum value at stage V testis at 460 dph (Figure 1C and Figure 2B). Notably, at 460 dph, no significant difference in GSI was observed between neomales and control males at stage V (Figure 2B,D).

Histological analysis was performed to classify the gonadal developmental stages of individual fish, followed by quantitative assessment of stage-specific frequencies at each sampling time point (Figure 2A). In control males, testes at 360 and 400 dph were predominantly at stages II and III (Figure 2A). At 430 dph, 25% of control testes had advanced to stage V, while the majority remained at stage IV (Figure 2A). At 460 dph, the majority of control testes (75%) had fully transitioned to stage V (Figure 2A). Neomales exhibited similar developmental patterns, with testes primarily at stages II and III at 360–400 dph (Figure 2A). At 430 dph, stage IV became the dominant developmental phase, although 25% of testes remained at stage II (Figure 2A). At 460 dph, most neomale testes (75%) progressed to stage V, while 25% were still at stage III (Figure 2A).

### 3.2. Gonadal Histology of Neomale Large Yellow Croaker

**Figure 3:**
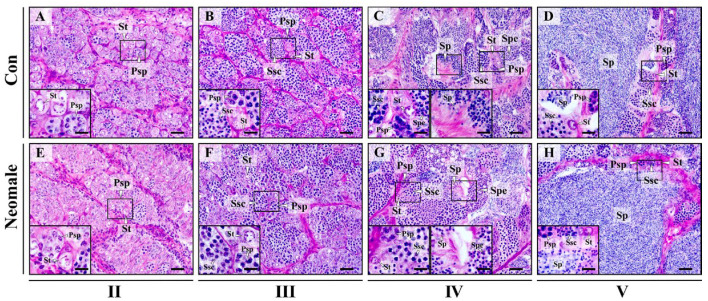
Histological examination of gonads in control males and neomales at distinct sampling timepoints (II: 360 dph, III: 400 dph, IV: 430 dph, V: 460 dph). Testes were categorized into four stages based on main germ cell: early spermatogenesis stage (II), late spermatogenesis stage (III), early spermiation stage (IV), late spermiation stage (V). (**A**) Control male fish testis in early spermatogenesis stage (II); (**B**) control male fish testis in late spermatogenesis stage (III); (**C**) control male fish testis in early spermiation stage (IV); (**D**) control male fish testis in late spermiation stage (V); (**E**) neomale fish testis in early spermatogenesis stage (II); (**F**) neomale fish testis in late spermatogenesis stage (III); (**G**) neomale fish testis in early spermiation stage (IV); (**H**) neomale fish testis in late spermiation stage (V). St = spermatogonia; Psp = primary spermatocyte; Ssc = secondary spermatocyte; Spe = spermatid; Sp = sperm. Scale bars = 20 µm (**A**–**H**), 10 µm (insert in (**A**–**H**)).

Histological studies were conducted on control males and neomales at different developmental stages (Figure 3). At 360 dph, we observed that the spermatogenic cells in stage II testes of both neomales and control males were predominantly spermatogonia, accompanied by a small number of primary spermatocytes. At 400 dph, the spermatogenic cells in stage III testes of neomales and control males were primarily spermatocytes, with a few spermatogonia localized at the periphery of the lobules. At 430 dph, in stage IV testes of neomales and control males, we observed a diverse array of spermatogenic cells, and the lobular lumens were filled with spermatids and sperm. At 460 dph, in stage V testes of neomales and control males, we observed abundant sperm, along with a small number of spermatogonia, primary spermatocytes, and secondary spermatocytes near the edges of the lobules, indicating that both neomales and control males had reached sexual maturity. Histological examination of neomales and control males across these developmental stages revealed no significant differences in gonadal development between the two groups.

### 3.3. Ultrastructural Observation of Spermatogenic Cells and Sertoli Cells in Neomale Large Yellow Croaker

**Figure 4:**
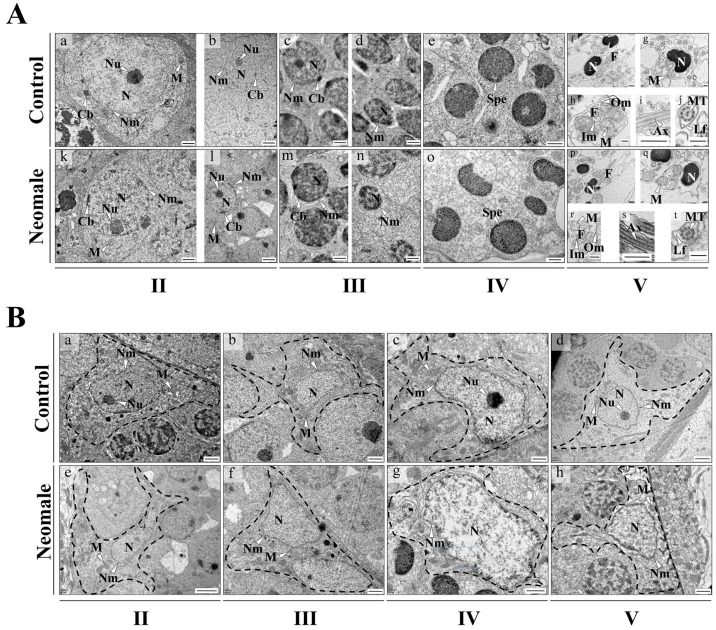
Ultrastructural examination of gonads in control males and pseudo-males across sampling timepoints (II: 360 dph, III: 400 dph, IV: 430 dph, V: 460 dph). (**Aa**) Primary spermatogonia in control male fish testis; (**Ab**) secondary spermatogonia in control male fish testis; (**Ac**) primary spermatocyte in control male fish testis; (**Ad**) secondary spermatocyte in control male fish testis; (**Ae**) spermatid in control male fish testis; (**Af**) longitudinal section of control male fish sperm; (**Ag**) cross-section of control male fish sperm nucleus; (**Ah**) cross-section through the midpiece of control male fish sperm; (**Ai**) longitudinal section through the flagellum of control male fish sperm; (**Aj**) cross-section through the flagellum of control male fish sperm; (**Ak**) primary spermatogonia in neomale fish testis; (**Al**) secondary spermatogonia in neomale fish testis; (**Am**) primary spermatocyte in neomale fish testis; (**An**) secondary spermatocyte in neomale fish testis; (**Ao**) spermatid in neomale fish testis; (**Ap**) longitudinal section of neomale fish sperm; (**Aq**) cross-section of neomale fish sperm nucleus; (**Ar**) cross-section through the midpiece of neomale fish sperm; (**As**) longitudinal section through the flagellum of neomale fish sperm; (**At**) cross-section through the flagellum of neomale fish sperm; (**Ba**–**Bd**) Sertoli cell in control male fish testis; (**Be**–**Bh**) Sertoli cell in neomale fish testis. Dashed boxes outline Sertoli cell ultrastructure. Cb = chromatoid body; N = nucleus; Nm = nuclear membrane; Nu = nucleolus; M = mitochondria; Spe = spermatid; Ax = axial filament; F = flagellum; Lf = lateral fin; Mt = microtubule; Im = inner membrane of sleeve; Om = outer membrane of sleeve. Scale bars = 200 nm (**Ag**,**Ah**,**Aj**,**Aq**,**At**), 500 nm (**Af**,**Ai**,**Ap**,**Ar**,**As**), 1 µm (**Aa**,**Ac**,**Ad**,**Ae**,**Ak**,**Am**,**An**,**Ao**,**Ba**,**Bb**,**Bc**,**Bf**,**Bg**,**Bh**), 2 µm (**Ab**,**Al**,**Bd**,**Be**).

Observations of germ cells and Sertoli cells in neomale and control male fish at distinct developmental stages were conducted via transmission electron microscopy (TEM) (Figure 4A). In the stage II testes of both 360 dph neomales and control males, two types of spermatogonia were observed. Primary spermatogonia, the largest among all spermatogenic cells, possess a large nucleus with a single, highly electron-dense nucleolus. The nuclear matrix and cytoplasm exhibit similar electron density (Figure 4(Aa,Ak)). The nuclear membrane displays a characteristic wavy double-layer structure with a clearly discernible intermembrane space. Near the nuclear membrane, chromatoid bodies composed of granular material are visible (Figure 4(Aa,Ak)). Secondary spermatogonia, derived from the division of primary spermatogonia, are smaller but similar in morphology to primary spermatogonia, with prominent nucleoli and chromatoid bodies distributed near the nuclear membrane (Figure 4(Ab,Al)). In the stage III testes of 400 dph neomales and control males, primary spermatocytes exhibit large nuclei with dispersed chromatin of varying shapes. Chromatoid bodies are still observable in the cytoplasm. Compared to primary spermatocytes, secondary spermatocytes show reduced nuclear volume and increased electron density, often eccentrically located (Figure 4(Ac,Ad,Am,An)). In the stage IV testes of 430 dph neomales and control males, the chromatin in metamorphic spermatids is highly condensed, leading to the disappearance of the electron density difference between the nuclear matrix and cytoplasm, resulting in blurred nuclear membrane boundaries, although the cell membrane retains a distinct double-layer structure (Figure 4(Ae,Ao)).

In the stage V testes of 460 dph neomales and control males, sperm consist of a head, midpiece, and tail (Figure 4(Af,Ap)). The cephalic architecture was fundamentally conserved between neomales and control males: an acrosome was absent at the anterior apex, while the reniform nucleus contained densely packed granular chromosomes with high electron density. Mitochondria were predominantly clustered at the nuclear periphery, exhibiting circular profiles in cross-sectional views (Figure 4(Ag,Ah,Aq,Ar)). The midpiece displayed a cylindrical sleeve enveloping the basal portion of the axoneme, with outer membranes demarcating mitochondrial boundaries. Spheroidal or elliptical mitochondria were discretely distributed around the axonemal base, arranged in concentric circular arrays. The tail exhibited a monoflagellar organization, with longitudinal sections revealing linear axonemes of high electron density (Figure 4(Ai,As)), while transverse sections demonstrated the canonical “9 + 2” microtubule configuration. The plasma membrane extended outwardly to form asymmetrically distributed lateral fins, manifesting as undulating short-fin projections in longitudinal sections (Figure 4(Aj,At)).

Sertoli cells in both neomales and control males exhibited similar ultrastructural features across all developmental stages: nuclei were large in volume with prominent double-layered nuclear membranes displaying widened intermembrane spaces (Figure 4(Ba–h)); the cytoplasm was enriched with oval, vesicular mitochondria (Figure 4(Ba–h)). Comprehensive TEM analysis revealed no significant ultrastructural differences in spermatogenic cells or Sertoli cells between neomales and control males at any developmental stage (Figure 4A,B).

### 3.4. Localization of Vasa, Sox9a and DMC1 Proteins in the Gonads of Neomale Large Yellow Croaker

**Figure 5:**
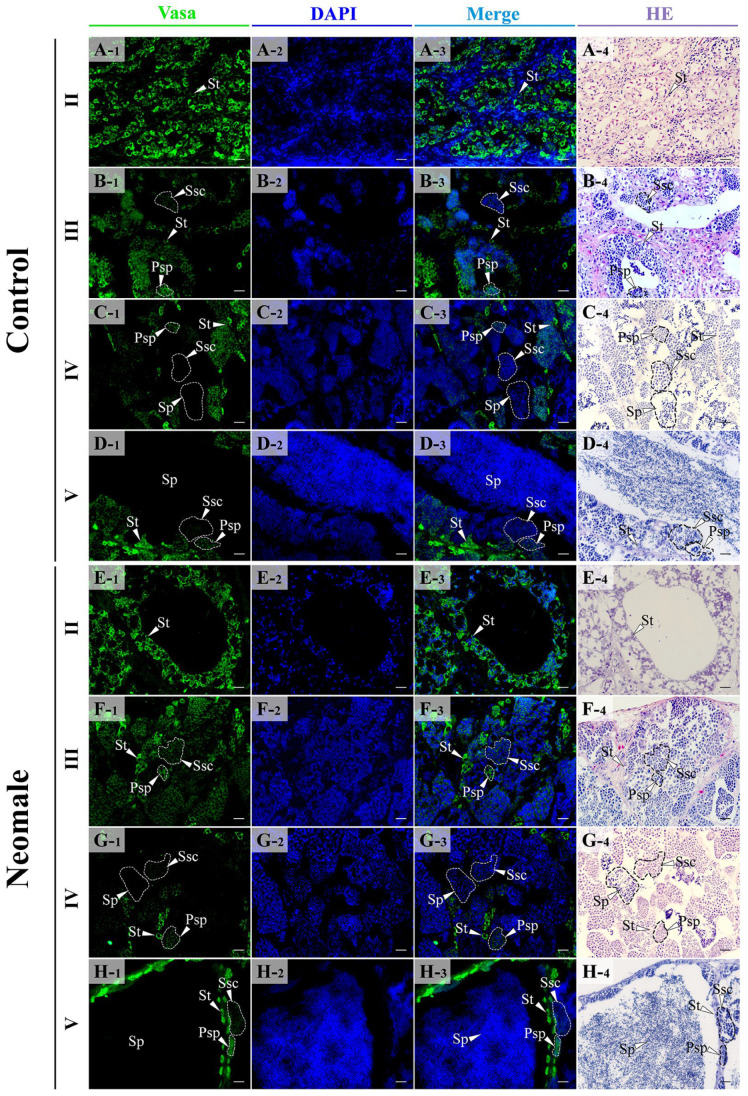
Location of Vasa protein in gonads for each developmental stage using immunofluorescent analysis. (**A1**–**A4**) Control male fish testis in early spermatogenesis stage (II); (**B1**–**B4**) control male fish testis in late spermatogenesis stage (III); (**C1**–**C4**) control male fish testis in early spermiation stage (IV); (**D1**–**D4**) control male fish testis in late spermiation stage (V); (**E1**–**E4**) neomale fish testis in early spermatogenesis stage (II); (**F1**–**F4**) neomale fish testis in late spermatogenesis stage (III); (**G1**–**G4**) neomale fish testis in early spermiation stage (IV); (**H1**–**H4**) neomale fish testis in late spermiation stage (V). Scale bars = 20 µm (**A1**–**H4**).

**Figure 6:**
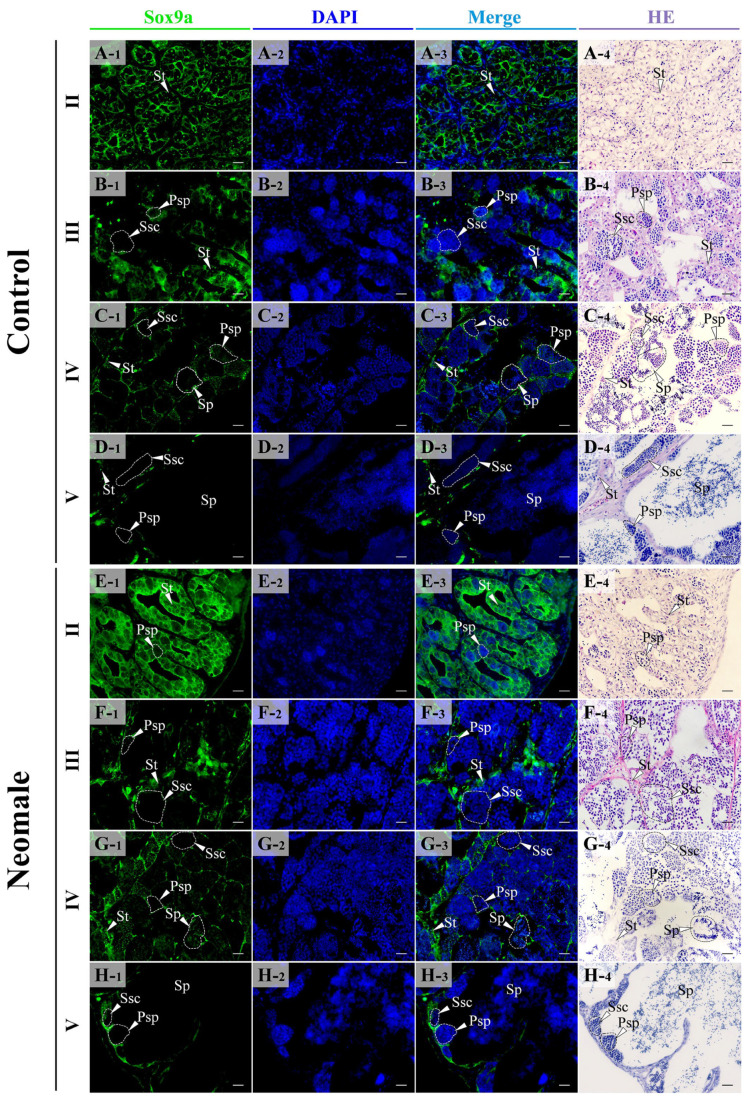
Location of Sox9a protein in gonads for each developmental stage using immunofluorescent analysis. (**A1**–**A4**) Control male fish testis in early spermatogenesis stage (II); (**B1**–**B4**) control male fish testis in late spermatogenesis stage (III); (**C1**–**C4**) control male fish testis in early spermiation stage (IV); (**D1**–**D4**) control male fish testis in late spermiation stage (V); (**E1**–**E4**) neomale fish testis in early spermatogenesis stage (II); (**F1**–**F4**) neomale fish testis in late spermatogenesis stage (III); (**G1**–**G4**) neomale fish testis in early spermiation stage (IV); (**H1**–**H4**) neomale fish testis in late spermiation stage (V). Scale bars = 20 µm (**A1**–**H4**).

**Figure 7:**
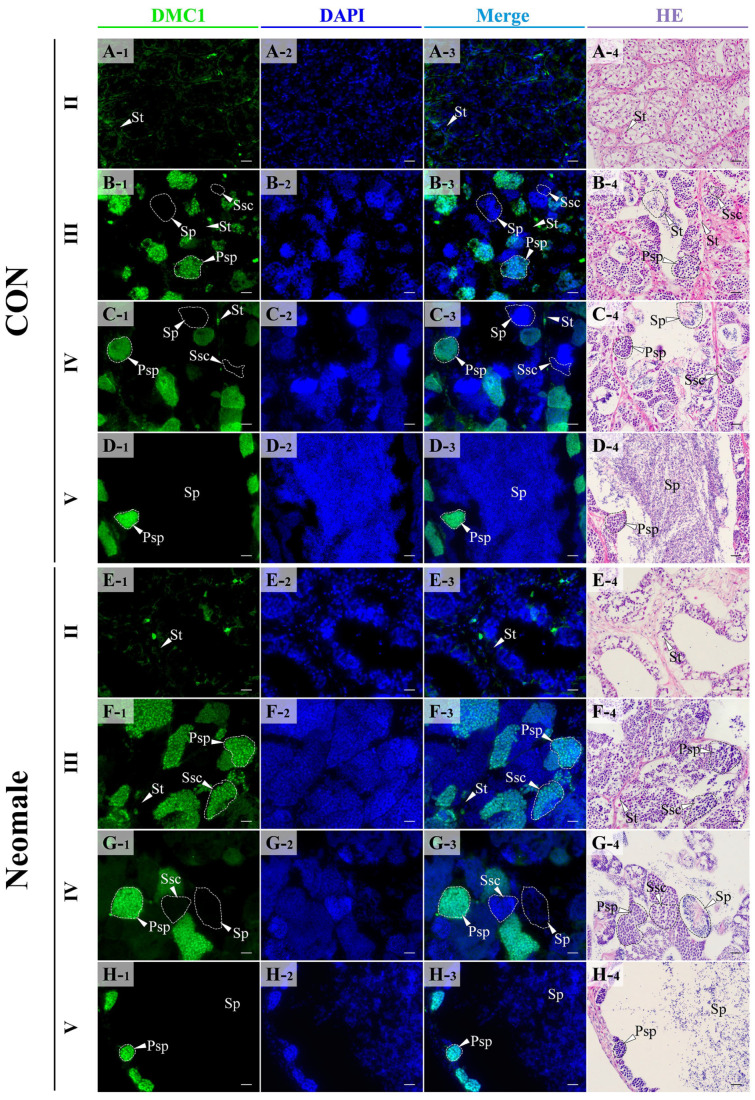
Location of DMC1 protein in gonads for each developmental stage using immunofluorescent analysis. (**A1**–**A4**) Control male fish testis in early spermatogenesis stage (II); (**B1**–**B4**) control male fish testis in late spermatogenesis stage (III); (**C1**–**C4**) control male fish testis in early spermiation stage (IV); (**D1**–**D4**) control male fish testis in late spermiation stage (V); (**E1**–**E4**) neomale fish testis in early spermatogenesis stage (II); (**F1**–**F4**) neomale fish testis in late spermatogenesis stage (III); (**G1**–**G4**) neomale fish testis in early spermiation stage (IV); (**H1**–**H4**) neomale fish testis in late spermiation stage (V). Scale bars = 20 µm (**A1**–**H4**).

The expression of Vasa, Sox9a, and DMC1 proteins in gonads at different developmental stages was detected by immunofluorescent observations (Figure 5, Figure 6 and Figure 7). In the testes of control males and neomales, Vasa protein was localized in the cytoplasm and was mainly expressed in spermatogonia, primary spermatocytes, and secondary spermatocytes (Figure 5A–H). In stage II testes, the fluorescence signal of Vasa was dense and concentrated in spermatogonia (Figure 5A,E). In stage III testes, the fluorescence signal of Vasa could be detected in spermatogonia, primary spermatocytes, and secondary spermatocytes (Figure 5B,F). In stage IV testes, the fluorescence signal was significantly weaker than that in stage III testes, and could only be detected in sparse spermatogonia and spermatocytes, with no signal observed in sperm (Figure 5C,G). In stage V testes, the signal density was low, and no signal could be observed on sperm, with only a few spermatogonia near the interlobular stroma showing signal (Figure 5D,H).

In the testes of control males and neomales, the Sox9a protein was localized in the cytoplasm, predominantly expressed in Sertoli cells and interstitial cells (Figure 6A–H). During the stage II testes, the fluorescence signal density of Sox9a was the most intense, being concentrated within the Sertoli cells (Figure 6A,E). Meanwhile, a faint signal was detected in the interstitial cells (Figure 6A,E). In stage III and stage IV testes, the signal density decreased (Figure 6B,C,F,G). In stage V testes, the signal density reached its lowest level, with the Sox9a protein primarily expressed in the Sertoli cells at the edge of the lobules and minimally in the interstitial cells (Figure 6D,H).

In the testes of control males and neomales, DMC1 protein was localized in the nucleus and was mainly expressed in primary spermatocytes (Figure 7A–H). In stage II testes, no fluorescence signal was detected in spermatogonia (Figure 7A,E). In stage III and IV testes, the signal was dense, and fluorescence signals could be detected in spermatocytes undergoing meiosis (Figure 7B,C,F,G). In stage V testes, the signal density decreased, and the signal only appeared in spermatocytes near the interlobular stroma, with no signal observed in sperm (Figure 7D,H).

## 4. Discussion

### 4.1. Growth and GSI Dynamics During Gonadal Maturation in Neomale Large Yellow Croaker

Neomales play a crucial role in the process of establishing all-female fish lines. Existing studies have confirmed that the administration of drugs and exogenous sex hormones during the undifferentiated stage of gonads in teleost fish can effectively induce sex reversal in fish [22]. Our research group successfully induced neomales in large yellow croaker by administering LTZ during the sensitive period of sex differentiation [9]. However, in fish, both aromatase inhibitor treatment and exogenous hormone administration have been found to exert adverse effects on induced neomales, including but not limited to growth anomalies, gonadal malformations, and reproductive dysfunction [14,15,23,24,25]. This study found the early LTZ treatment exerted no significant impact on growth during early stage of large yellow croaker. Previous reports show inconsistent growth effects of LTZ across fish species: Low-dose LTZ exposure enhanced early growth in yellow catfish, though this effect disappeared after withdrawal [14]. In contrast, rosy barb exhibited growth suppression during LTZ treatment [16]. Notably, we observed significantly higher GSI in 400 and 430 dph neomales compared to control males. Aromatase inhibitors were likely to exert differential effects on the GSI across species. For example, in dwarf gourami, aromatase inhibition increased GSI and spermatogenic cell density, whereas in rosy barb, it decreased GSI despite elevating spermatogenic cell counts, indicating no direct correlation between GSI fluctuations and spermatogenic cell density [16,26]. In three-spot wrasse, aromatase inhibitors suppressed spermatogonial proliferation, resulting in reduced GSI [17]. Conversely, early aromatase inhibition in European sea bass altered estrogen functionality, exerting long-term effects on testicular estrogen activity and ultimately elevating GSI [19].

### 4.2. Testicular Histological and Ultrastructural Integrity with Spermatogenic Synchrony in Neomale Large Yellow Croaker

Histological results showed no abnormalities in the structure of neomale testes or in the morphology and distribution of spermatogenic cells across developmental stages (Figure 3E–H). Further transmission electron microscopy (TEM) observations demonstrated progressive nuclear condensation during spermatogenesis, a process essential for cellular maturation and transition to subsequent stages, consistent with the general pattern of nuclear-cytoplasmic remodeling in fish. Our findings align with previous reports in yellow catfish, where aromatase inhibitor-induced neomales exhibited unimpaired spermatogenesis culminating in functional sperm production [14]. However, studies on hybrid red tilapia masculinization and dwarf gourami documented aromatase inhibitor-induced abnormalities, including Leydig cell hypertrophy, disrupted spermatogenesis, and altered spermatogenic cell density [15,26]. These interspecific discrepancies may stem from differential sensitivity to aromatase inhibitors. Testicular maintenance requires basal estradiol levels, and aromatase inhibition-induced estrogen reduction could impair gonadal development [15,27,28]. Notably, LTZ-treated neomales of large yellow croaker displayed intact testicular architecture and spermatogenic cell morphology, completing full spermatogenesis.

Sperm ultrastructure, a critical indicator of sperm quality, directly influences motility, fertilization capacity, and consequently the efficiency of all-female population establishment [29,30,31]. TEM analysis revealed highly conserved ultrastructural features in neomale sperm: chromatin in the head exhibited typical high-electron-density granular arrangement (Figure 4(Ae,An)), mitochondria formed a ring-like distribution in the neck region (Figure 4(Af,Ao)), and the flagellar axoneme displayed the standard “9 + 2” microtubule configuration (Figure 4(Ah,Aq)), showing no significant differences from control males (Figure 4A,B). In contrast, neomales of topmouth culter exhibited ultrastructural defects, including mitochondrial damage and membrane swelling, which compromised sperm quality [32]. These results indicate that sex reversal did not compromise sperm structural integrity in large yellow croaker neomales, though further analyses of sperm motility, fertilization rate, and hatching success are required to fully assess their reproductive performance.

Our study demonstrates that the developmental stage proportions of testes in neomales closely matched those of control males across sampling timepoints. Importantly, neomales of large yellow croaker showed no signs of premature sexual maturation during spermatogenesis. The high developmental synchrony between neomales and control males in spermatogenic progression provides crucial assurance for their reliability as broodstock during spawning seasons.

### 4.3. Expression Patterns of vasa, sox9a, and dmc1 During Spermatogenesis in Neomale Large Yellow Croaker

Vasa protein, a crucial member of the DEAD-box protein family, serves as one of the key regulatory factors determining germline development [33]. The *vasa* gene has been widely recognized as an ideal molecular marker for primordial germ cells (PGCs) and germ cells, facilitating the investigation of PGCs’ origin, migration, and differentiation pathways [34]. Current evidence confirms that *vasa* expression is exclusively localized in germ cells of fish gonads [33]. In this study, we observed Vasa protein expression in spermatogonia and spermatocytes within testicular tissues, with spermatogonia exhibiting strong fluorescent signals while spermatocytes showed weaker signals. Of particular note, no detectable signals were observed in spermatozoa. This expression pattern aligns with previous reports in zebrafish, guppy, and yellow drum [35,36,37,38]. Previous studies have demonstrated *vasa*’s essential role in ensuring proper spermatogenesis and sperm production, with its expression being susceptible to pharmaceutical and hormonal interventions [39,40,41]. Our quantitative real-time PCR (qPCR) results revealed similar expression trends of the *vasa* gene in neomales and control males, showing no significant differences in expression levels (Figure S1). Therefore, we speculated that early LTZ treatment did not alter *vasa*’s expression pattern, suggesting normal migration and differentiation of germ cells during development.

As a multifunctional transcription factor in the SOX protein family, Sox9a mediates androgen signaling regulation through binding with the androgen receptor (AR) during spermatogenesis, thereby activating the transcription of the anti-Müllerian hormone (*amh*) gene [42,43]. Although *sox9a* exhibits broad expression across multiple tissues including brain, kidney, muscle, testis, and intestines, its protein expression pattern remains undefined in teleosts [44]. This study revealed predominant localization of Sox9a protein in Sertoli cells during various spermatogenic stages, with weak signals detected in surrounding Leydig cells and spermatogenic cells. This finding shows subtle differences from previous reports showing exclusive Sertoli cell localization in zebrafish testis [43]. Notably, the exosome-multivesicular body pathway enables Sertoli cells to transport proteins and nucleic acids to spermatogenic cells to maintain spermatogenic homeostasis, while exosome-mediated communication with Leydig cells regulates testosterone synthesis [13], potentially explaining the weak signals in spermatogenic cells. Previous studies demonstrate that *sox9a* plays crucial roles in maintaining spermatogenic cell homeostasis, regulating differentiation, and controlling cell quantity, directly influencing testicular development in fish [45,46,47]. The qPCR analysis demonstrated significantly elevated *sox9a* expression in stage V testes of neomales, which may be associated with gonad maturation, maintenance of spermatogenic cell quantity, and testicular structural stability in neomales (Figure S2). Remarkably, the highly conserved expression patterns between neomales and normal males suggest that sex reversal processes do not compromise Sertoli cell differentiation or functional integrity, thereby ensuring testicular structural stability and supporting meiotic progression of spermatogenic cells.

DMC1 is a highly conserved meiotic recombinase in eukaryotes, responsible for promoting homologous chromosome recombination and synapsis [48,49]. Furthermore, Dmc1 serves as a molecular marker for human spermatocytes at leptotene to zygotene stages [50]. In this study, DMC1 protein was predominantly localized in primary spermatocytes, with no detectable signals observed in spermatogonia, secondary spermatocytes, or spermatozoa. These findings align with previous reports in Japanese eel, where DMC1 signals were exclusively detected in primary spermatocytes [48]. In fish, *dmc1* is predominantly expressed in gonads and exhibits high evolutionary conservation [51]. Proper expression of *dmc1* is essential for maintaining meiotic homeostasis, and its expression dynamics can serve as a marker for germ cell entry into meiosis [48]. For instance, *dmc1* mutation in medaka results in meiotic defects and abnormal sperm morphology [52]. The qPCR analysis revealed consistent *dmc1* expression trends between neomales and control males across developmental stages, with no significant differences in expression levels (Figure S3). Neomales and control males showed identical *dmc1* expression patterns, suggesting normal meiotic progression in the present study.

## 5. Conclusions

This study systematically analyzed the spermatogenesis process in neomales, with emphasis on gonadal histology and expression patterns of key proteins during spermatogenesis. Although we observed temporal divergence in gonadal development between two groups, neomales exhibited similar growth patterns and testicular microstructure to control males. Immunofluorescence results further confirmed the conserved spatiotemporal localization of critical proteins involved in spermatogenesis. Collectively, the spermatogenic progression in neomales closely resembles that of control males, ensuring their reliability as broodstock parents. These findings establish a theoretical foundation and provide technical support for large-scale establishment of all-female fish populations.

## Data Availability

The original contributions presented in this study are included in the article and Supplementary Materials. Further inquiries can be directed to the corresponding author.

## Supplementary Materials

The following supporting information can be downloaded at: https://www.mdpi.com/article/10.3390/biology14081054/s1, Figure S1: Relative expression of *vasa* in gonads of control male and neomale large yellow croaker; Figure S2: Relative expression of *sox9a* in gonads of control male and neomale large yellow croaker; Figure S3: Relative expression of *dmc1* in gonads of control male and neomale large yellow croaker; Table S1: Primer sequences; Table S2: Sampling information record.

## References

[1] The Bureau of Fisheries and Fishery Administration, the Ministry of Agriculture (2024). China Fishery Statistical Yearbook.

[2] Liu H., Fan S., Xu Q., Wang X., Zhang Y., Chen W., Hu Y., Deng X., Liu H., Yang C. (2025). Germplasm innovation of large yellow croaker and its research progress. Reprod. Breed..

[3] Wang Z., Cai M. (2018). Artificial Gynogenesis and Sex Control in Large Yellow Croaker. Sex Control in Aquaculture.

[4] You X., Cai M., Jiang Y., Wang Z. (2012). Histological observation on gonadal sex differentiation in large yellow croaker (*Larimichthys crocea*). J. Fish. China.

[5] Xu J., You F., Yan B., Zhang P. (2007). Effects of ultra-violet irradiation on sperm motility and diploid gynogenesis induction in large yellow croaker (*Pseudosciaena crocea*) undergoing cold shock. Aquac. Int..

[6] Cai M., Wu Q., Liu X., Yao C., Chen Q., Wang Z. (2010). Artificial induction of mito-gynogenetic diploids in large yellow croaker (*Pseudosciaena crocea*) by hydrostatic pressure. Chin. J. Oceanol. Limnol..

[7] Yu M., Xie Q., Wei F., Wu X., Xu W.-T., Zhan W., Liu F., Guo D.-D., Niu B.-L., Lou B. (2022). Development and identification of a sex-specific molecular marker in Dai-qu stock large yellow croaker (*Larimichthys crocea*). Aquaculture.

[8] Lin A., Xiao S., Xu S., Ye K., Lin X., Sun S., Wang Z. (2017). Identification of a male-specific DNA marker in the large yellow croaker (*Larimichthys crocea*). Aquaculture.

[9] Mu J., Hu W., Chen R., Yang Y., Li H., Li W., Yin X., Xu D. (2024). Production of neomale and neofemale large yellow croaker (*Larimichthys crocea*) and establishment of all-female populations. Aquaculture.

[10] Piferrer F., Lee C.-S., Donaldson E.M. (2001). Endocrine Sex Control Strategies for the Feminization of Teleost Fish. Reproductive Biotechnology in Finfish Aquaculture.

[11] Qin Z., Yang F., Tian L., Chen R., Xu D., Takeuchi Y. (2019). Induction of sex reversal in blue drum (*Nibea mitsukurii*) and gynogenetic yellow drum (*Nibea albiflora*) by oral administration of letrozole. Aquac. Res..

[12] Schulz R.W., de França L.R., Lareyre J.-J., LeGac F., Chiarini-Garcia H., Nobrega R.H., Miura T. (2010). Spermatogenesis in fish. Gen. Comp. Endocrinol..

[13] Xiao Y., Zhang J., Guan Y., Wang M., Liu D., Xiong S., Li J., Yu X. (2025). Research progress on Sertoli cell secretion during spermatogenesis. Front. Endocrinol..

[14] Shen Z., Fan Q., Yang W., Zhang Y., Wang H. (2015). Effects of 17a-Methyltestosterone and Aromatase Inhibitor Letrozole on Sex Reversal, GonadalStructure, and Growth in Yellow Catfish Pelteobagrusfulvidraco. Biol. Bull..

[15] Syanya F.J., Mahadevan H., Khanna A.R.N. (2025). The effects of a non-steroid aromatase inhibitor on hybrid red tilapia masculinization, growth, reproductive hormone profile, and economic efficiency in aquaculture. Aquac. Int..

[16] Meena D.K., Lal J., Biswas P., Singh S.K., Debbarma R., Deb S., Yadav N.K., Patel A.B. (2023). Effects of dietary aromatase inhibitors on masculinization of rosy barb (*Pethia conchonius*): Evidence from growth, coloration and gonado-physiological changes. PLoS ONE.

[17] Kobayashi Y., Nozu R., Nakamura M. (2010). Role of estrogen in spermatogenesis in initial phase males of the three-spot wrasse (*Halichoeres trimaculatus*): Effect of aromatase inhibitor on the testis. Dev. Dyn..

[18] Singh A.K., Srivastava P.P., Verma R., Srivastava S.C., Kumar D., Ansari A. (2015). Effect of dietary administration of letrozole and tamoxifen on gonadal development, sex differentiation and biochemical changes in common carp (*Cyprinus carpio* L.). Reprod. Fertil. Dev..

[19] Navarro-Martín L., Blázquez M., Piferrer F. (2009). Masculinization of the European sea bass (*Dicentrarchus labrax*) by treatment with an androgen or aromatase inhibitor involves different gene expression and has distinct lasting effects on maturation. Gen. Comp. Endocrinol..

[20] Breton T.S., Kenter L.W., Greenlaw K., Montgomery J., Goetz G.W., Berlinsky D.L., Luckenbach J.A. (2019). Initiation of sex change and gonadal gene expression in black sea bass (*Centropristis striata*) exposed to exemestane, an aromatase inhibitor. Comp. Biochem. Phys. A.

[21] Nakamura M. (2013). Morphological and Physiological Studies on Gonadal Sex Differentiation in Teleost Fish. Aqua-Biosci. Monogr..

[22] Budd A., Banh Q., Domingos J., Jerry D. (2015). Sex Control in Fish: Approaches, Challenges and Opportunities for Aquaculture. J. Mar. Sci. Eng..

[23] Tsumura K., Blann V.E., Lamont C.A. (1991). Progeny Test of Masculinized Female Rainbow Trout Having Functional Gonoducts. Progress. Fish-Cult..

[24] Chatain B., Saillant E., Peruzzi S. (1999). Production of monosex male populations of European seabass, Dicentrarchus labrax L. by use of the synthetic androgen 17α-methyldehydrotestosterone. Aquaculture.

[25] Xu D., Yang F., Chen R., Lou B., Zhan W., Hayashida T., Takeuchi Y. (2018). Production of neo-males from gynogenetic yellow drum through 17α-methyltestosterone immersion and subsequent application for the establishment of all-female populations. Aquaculture.

[26] Katare M.B., Pai M., HS M. (2021). Effect of anastrozole on masculinization in ornamental fish, dwarf gourami, *Trichogaster lalius* (Hamilton, 1822). Indian J. Exp. Biol..

[27] Lazier C.B., Langley S., Ramsey N.B., Wright J.M. (1996). Androgen inhibition of Vitellogenin Gene Expressionin Tilapia (*Oreochromis niloticus*). Gen. Comp. Endocrinol..

[28] Kim K., Moon H., Yeo I. (2023). Inhibition of sexual maturation inhibition using exemestane and tamoxifen in female olive flounder (*Paralichthys olivaceous*). Res. Sq..

[29] Malo A.F., Gomendio M., Garde J., Lang-Lenton B., Soler A.J., Roldan E.R.S. (2006). Sperm design and sperm function. Biol. Lett..

[30] Guo W., Shao J., Li P., Wu J., Wei Q. (2016). Morphology and ultrastructure of *Brachymystax lenok tsinlingensis* spermatozoa by scanning and transmission electron microscopy. Tissue Cell.

[31] Anderson M.J., Dixson A.F. (2002). Motility and the midpiece in primates. Nature.

[32] Jia Y. (2019). Topmouth Culter (*Culter alburnus*) All-Female Breedingsystem Establishment and Evaluation. Ph.D. Thesis.

[33] Begum S., Gnanasree S.M., Anusha N., Senthilkumaran B. (2022). Germ cell markers in fishes—A review. Aquac. Fish..

[34] Yoon C., Kawakami K., Hopkins N. (1997). Zebrafish *vasa* homologue RNA is localized to the cleavage planes of 2- and 4-cell-stage embryos and is expressed in the primordial germ cells. Development.

[35] Braat A.K., van de Water S., Goos H., Bogerd J., Zivkovic D. (2000). Vasa protein expression and localization in the zebrafish. Mech. Dev..

[36] Milani L., Cinelli F., Iannello M., Lazzari M., Franceschini V., Maurizii M.G. (2022). Immunolocalization of Vasa, PIWI, and TDRKH proteins in male germ cells during spermatogenesis of the teleost fish *Poecilia reticulata*. Acta Histochem..

[37] Yu L., Yang Y., Yu Y., Li H., Chen R., Miao L., Xu D. (2024). Gametogenesis and *vasa* expression are seasonally regulated in yellow drum (*Nibea albiflora*). Aquac. Rep..

[38] Yang Y., Lu L., Chen R., Yu L., Hu W., Xu D. (2023). Production of sterile mono-sex triploid yellow drum (*Nibea albiflora*): Genotypic females and sex-reversed phenotypic males with emphasis on utilization as surrogate broodstock. Fish Physiol. Biochem..

[39] Tanaka S.S., Toyooka Y., Akasu R., Katoh-Fukui Y., Nakahara Y., Suzuki R., Yokoyama M., Noce T. (2000). The mouse homolog of Drosophila *Vasa* is required for the development of male germ cells. Gene Dev..

[40] Cardinali M., Gioacchini G., Candiani S., Pestarino M., Yoshizaki G., Carnevali O. (2004). Hormonal regulation of *vasa*-like messenger RNA expression in the ovary of the marine teleost *Sparus aurata*. Biol. Reprod..

[41] Hartung O., Forbes M.M., Marlow F.L. (2014). Zebrafish vasa is required for germ-cell differentiation and maintenance. Mol. Reprod. Dev..

[42] Zhai G., Shu T., Xia Y., Jin X., He J., Yin Z. (2017). Androgen signaling regulates the transcription of anti-Mullerian hormone via synergy with SRY-related protein SOX9A. Sci. Bull..

[43] Rodríguez-Marí A., Yan Y.-L., BreMiller R.A., Wilson C., Cañestro C., Postlethwait J.H. (2005). Characterization and expression pattern of zebrafish anti-Müllerian hormone (*amh*) relative to *sox9a*, *sox9b*, and *cyp19a1a*, during gonad development. Gene Expr. Patterns.

[44] Zhang M., Zhu Y., Li W., Shen W., Wu X., Ye K., Wang Z. (2019). Cloning and expression of *sox9a/b* gene in the large yellow croaker (*Larimichthys crocea*). J. Fish. China.

[45] Li X.Y., Tang Y.H., Deng W.Y., Zheng Y., Wang L.S., He X., Xie Q.P., Li Y.Q., Deng L., Wang D.S. (2023). Involvement of Sox9a in chondrogenesis and gonadal development in teleost Nile tilapia (*Oreochromis niloticus*). Zool. Res..

[46] Nakamura S., Watakabe I., Nishimura T., Toyoda A., Taniguchi Y., Tanaka M. (2012). Analysis of Medaka sox9 Orthologue Reveals a Conserved Role in Germ Cell Maintenance. PLoS ONE.

[47] Symon A., Harley V. (2017). SOX9: A genomic view of tissue specific expression and action. Int. J. Biochem. Cell Biol..

[48] Kajiura-Kobayashi H., Kobayashi T., Nagahama Y. (2005). Cloning of cDNAs and the differential expression of A-type cyclins and Dmc1 during spermatogenesis in the Japanese eel, a teleost fish. Dev. Dyn..

[49] Xu J., Zhao L., Peng S., Chu H., Liang R., Tian M., Connell P.P., Li G., Chen C., Wang H. (2021). Mechanisms of distinctive mismatch tolerance between Rad51 and Dmc1 in homologous recombination. Nucleic Acids Res..

[50] El-Behery E.I., El-Naseery N.I., El-Ghazali H.M., Elewa Y.H.A., Mahdy E.A.A., El-Hady E., Konsowa M.M.H. (2019). The efficacy of chronic zinc oxide nanoparticles using on testicular damage in the streptozotocin-induced diabetic rat model. Acta Histochem..

[51] Tao M., Liu S., Long Y., Zeng C., Liu J., Liu L., Zhang C., Duan W., Liu Y. (2008). The cloning of *Dmc1* cDNAs and a comparative study of its expression in different ploidy cyprinid fishes. Sci. China Ser. C.

[52] Chen J., Cui X., Jia S., Luo D., Cao M., Zhang Y., Hu H., Huang K., Zhu Z., Hu W. (2016). Disruption of *dmc1* Produces Abnormal Sperm in Medaka (*Oryzias latipes*). Sci. Rep.

