# Supplementary material for: Functional Spermatogenesis Across Testicular Developmental Stages in Neomale Large Yellow Croaker (Larimichthys crocea) Revealed by Histology and Gonadal Specific Cellular Markers
Source: Biology (Basel). 2025 Aug 14;14(8):1054. doi: 10.3390/biology14081054 (PMC12383341; doi:10.3390/biology14081054)
Supplement: Supplementary file 1 [file biology-14-01054-s001.zip › Table S2.pdf]

Table S2: Sampling Information Record				
**Days post-hatch (dph)**	**Sample size Control male(XY, ♂)/Neomale(XX,♂)**	**Sampled tissue**	**Preservation reagent**	**Experimental method**
150	15/15	-	-	Body length and body weight measurement
		Fin clip	Absolute ethanol	DNA extraction
		Gonad (Part A)	Bouin's Fixative Solution	Histological analysis
180	15/15	-	-	Body length and body weight measurement
		Fin clip	Absolute ethanol	DNA extraction
		Gonad (Part A)	Bouin's Fixative Solution	Histological analysis
240	15/15	-	-	Body length and body weight measurement
		Fin clip	Absolute ethanol	DNA extraction
		Gonad (Part A)	Bouin's Fixative Solution	Histological analysis
300	15/15	-	-	Body length and body weight measurement
		Fin clip	Absolute ethanol	DNA extraction
		Gonad (Part A)	Bouin's Fixative Solution	Histological analysis
360	8/8	Gonad (Part A)	Bouin's Fixative Solution	Histological analysis
		Gonad (Part B)	4% Paraformaldehyde Fixative	Immunofluorescent analysis
		Gonad (Part C)	RNA wait	Quantitative real-time PCR (qPCR)
		Gonad (Part D)	2.5% glutaraldehyde Fixative	Transmission electron microscopy (TEM) analysis
		-	-	Body length, body weight and gonad weight measurement
		Fin clip	Absolute ethanol	DNA extraction
400	8/8	Gonad (Part A)	Bouin's Fixative Solution	Histological analysis
		Gonad (Part B)	4% Paraformaldehyde Fixative	Immunofluorescent analysis
		Gonad (Part C)	RNA wait	Quantitative real-time PCR (qPCR)
		Gonad (Part D)	2.5% glutaraldehyde Fixative	Transmission electron microscopy (TEM) analysis
		-	-	Body length, body weight and gonad weight measurement
		Fin clip	Absolute ethanol	DNA extraction
430	8/8	Gonad (Part A)	Bouin's Fixative Solution	Histological analysis
		Gonad (Part B)	4% Paraformaldehyde Fixative	Immunofluorescent analysis
		Gonad (Part C)	RNA wait	Quantitative real-time PCR (qPCR)
		Gonad (Part D)	2.5% glutaraldehyde Fixative	Transmission electron microscopy (TEM) analysis
		-	-	Body length, body weight and gonad weight measurement
		Fin clip	Absolute ethanol	DNA extraction
460	8/8	Gonad (Part A)	Bouin's Fixative Solution	Histological analysis
		Gonad (Part B)	4% Paraformaldehyde Fixative	Immunofluorescent analysis
		Gonad (Part C)	RNA wait	Quantitative real-time PCR (qPCR)
		Gonad (Part D)	2.5% glutaraldehyde Fixative	Transmission electron microscopy (TEM) analysis
		-	-	Body length, body weight and gonad weight measurement
		Fin clip	Absolute ethanol	DNA extraction

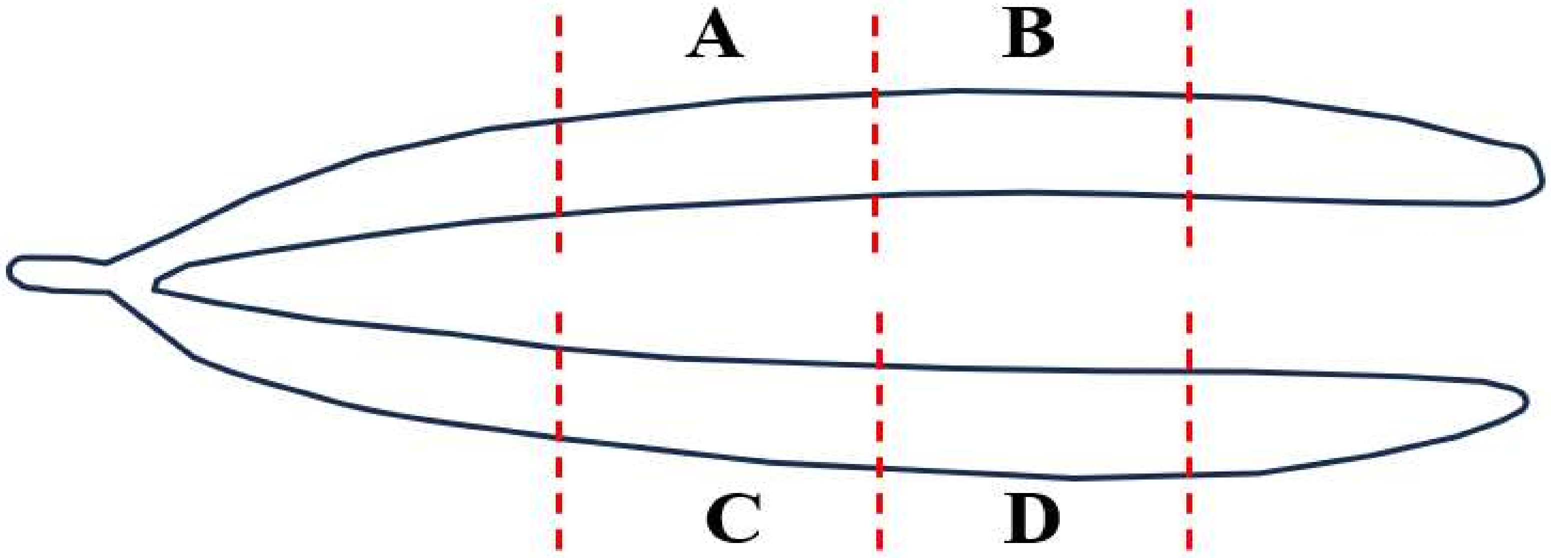

Figure: Schematic Diagram of Gonad Partitioning
