# Supplementary figures and images for: Functional Spermatogenesis Across Testicular Developmental Stages in Neomale Large Yellow Croaker (Larimichthys crocea) Revealed by Histology and Gonadal Specific Cellular Markers
Source: Biology (Basel). 2025 Aug 14;14(8):1054. doi: 10.3390/biology14081054 (PMC12383341; doi:10.3390/biology14081054)

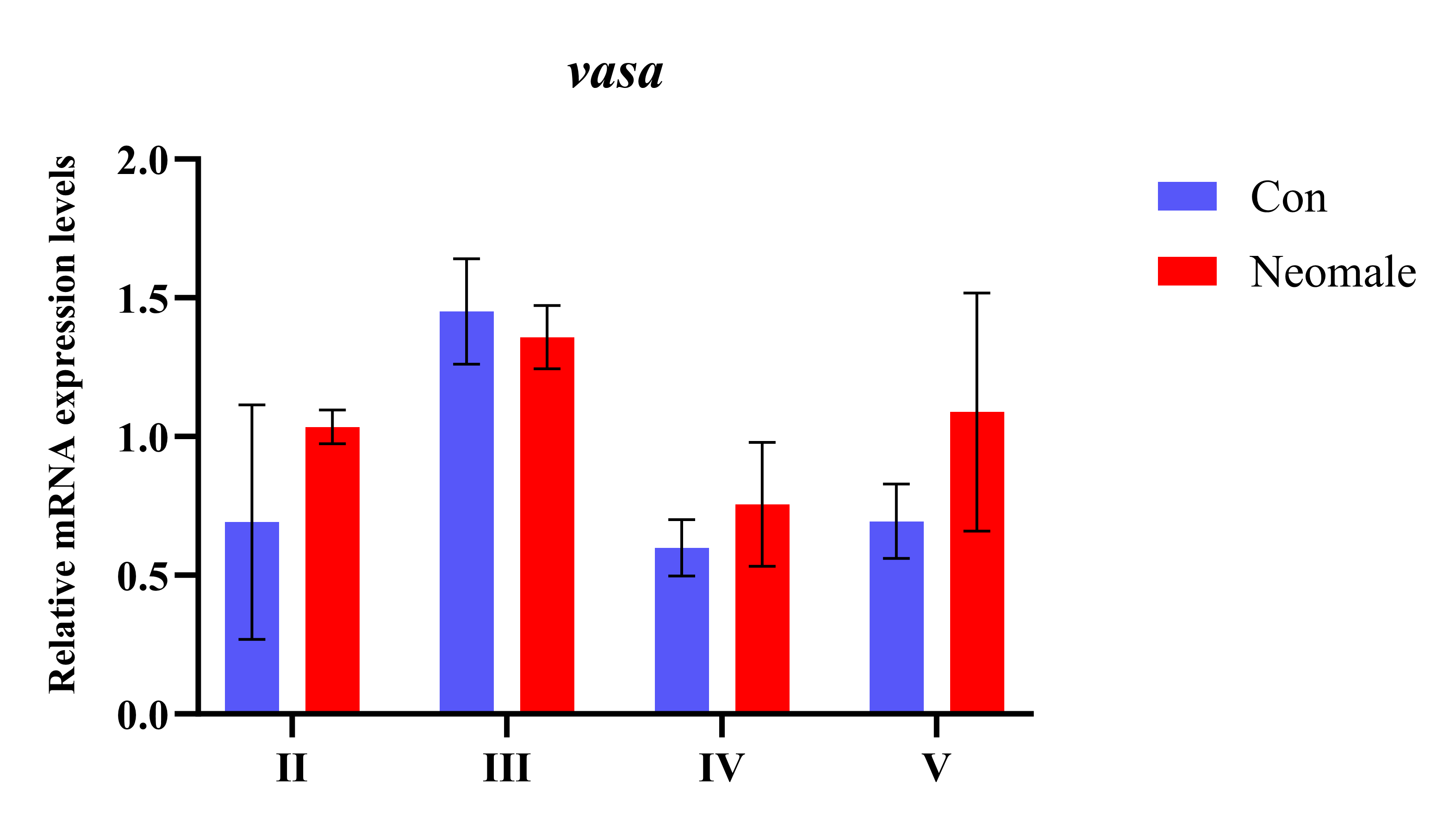

Supplement: Supplementary file 1 [file biology-14-01054-s001.zip › Figure S1.tif]

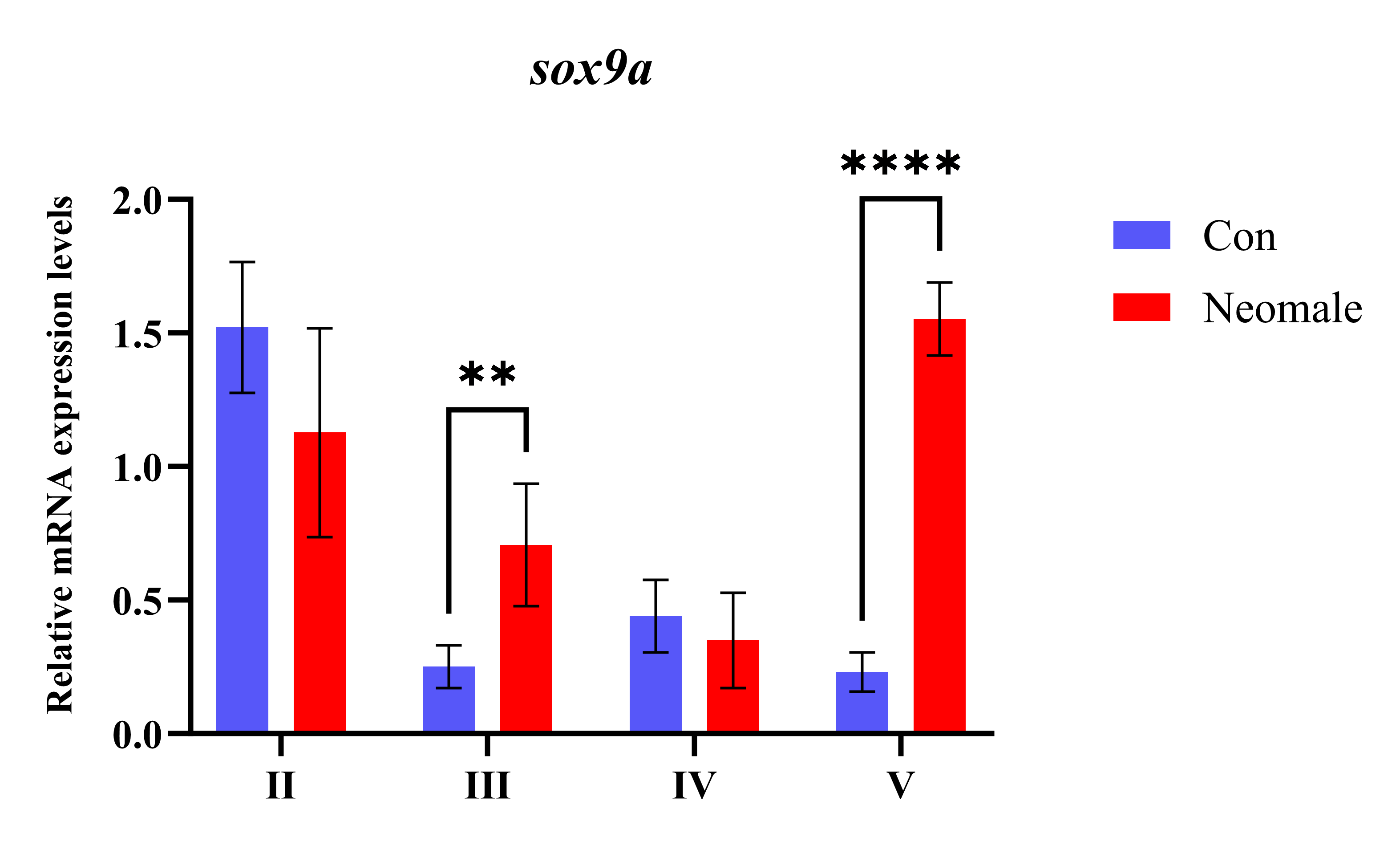

Supplement: Supplementary file 1 [file biology-14-01054-s001.zip › Figure S2.tif]

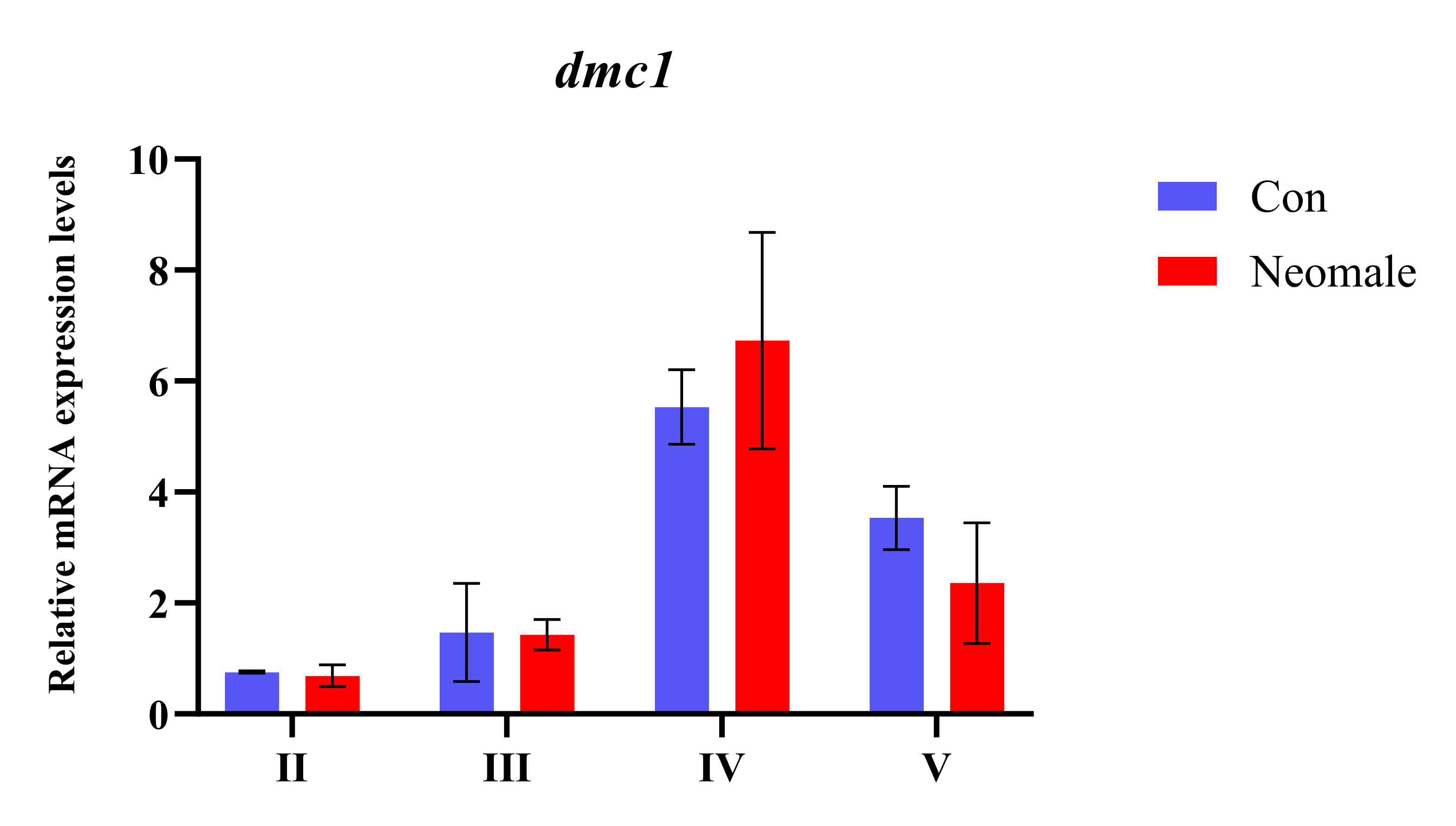

Supplement: Supplementary file 1 [file biology-14-01054-s001.zip › Figure S3.tif]
